# Limited directed seed dispersal in the canopy as one of the determinants of the low hemi-epiphytic figs’ recruitments in Bornean rainforests

**DOI:** 10.1371/journal.pone.0217590

**Published:** 2019-06-13

**Authors:** Miyabi Nakabayashi, Yoichi Inoue, Abdul Hamid Ahmad, Masako Izawa

**Affiliations:** 1 Graduate School of Engineering and Science, University of the Ryukyus, Senbaru, Nishihara, Okinawa, Japan; 2 The School of Arts and Sciences, The University of Tokyo, Komaba, Tokyo, Japan; 3 Institute for Tropical Biology and Conservation, Universiti Malaysia Sabah, Jalan UMS, Kota Kinabalu, Sabah, Malaysia; 4 Faculty of Science, University of the Ryukyus, Senbaru, Nishihara, Okinawa, Japan; University of Nottingham Malaysia Campus, MALAYSIA

## Abstract

*Ficus* species are keystone plants in tropical rainforests, and hemi-epiphytic figs play a notably important role in forest ecosystems. Because hemi-epiphytic figs have strict germination requirements, germination and establishment stages regulate their populations. Despite the ecological importance of hemi-epiphytic figs in the rainforests, seed dispersal systems by fig-eating animals under natural conditions remain unknown because of the difficulty in tracing the destiny of dispersed seeds in the canopy. Therefore, seed dispersal effectiveness (SDE) has never been evaluated for hemi-epiphytic figs. We evaluated the SDE of hemi-epiphytic figs using qualitative and quantitative components by three relatively large-sized (> 3 kg) arboreal and volant animals in Bornean rainforests that largely depend on fig fruits in their diets: binturongs *Arctictis binturong*, Mueller’s gibbons *Hylobates muelleri*, and helmeted hornbills *Rhinoplax vigil*. The SDE values of binturongs was by far the highest among the three study animals. Meanwhile, successful seed dispersal of hemi-epiphytic figs by gibbons and helmeted hornbills is aleatory and rare. Given that seed deposition determines the fate of hemi-epiphytic figs, the defecatory habits of binturongs, depositing feces on specific microsites in the canopy, is the most reliable dispersal method, compared to scattering feces from the air or upper canopy. We showed that reliable directed dispersal of hemi-epiphytic figs occurs in high and uneven canopy of Bornean rainforests. This type of dispersal is limited to specific animal species, and therefore it may become one of the main factors regulating low-success hemi-epiphytic fig recruitment in Bornean rainforests.

## Introduction

*Ficus* (Moraceae) is one of the world’s largest woody plant genera with approximately 750 species of various growth forms: trees, shrubs, climbers, epiphytes, and hemi-epiphytes [1]. *Ficus* species are distributed pantropically, but the majority are found in Malesia and Australia [2]. Each individual produces ripe fig fruits asynchronously and aseasonally; therefore, each fig population exhibits continual fruiting throughout the year [3]. This fruiting pattern enables obligate pollination mutualism with wasps (Agaonidae) by maintaining the pollinating wasp population [4]. Moreover, because of their year-round fruiting patterns, *Ficus* species are considered keystone food resources for animals in tropical rainforests, especially when the availability of preferred fruits is low [3,5–7]. Although this usually applies to whole *Ficus* communities, *Ficus* species vary considerably in reproductive system, growth form, frugivore guild (e.g. generalist and specialist), and habitat [6]. Among the *Ficus* species, hemi-epiphytes include approximately 300 species [1] and occupy one-third to over one-half of the fig species in the rainforest community [8]. They are a major component of canopy ecosystems across tropical regions [9], and they increase population turnover and forest regeneration by causing host tree-fall [10]. Therefore, hemi-epiphytic figs play a notably important role in forest ecosystems.

Hemi-epiphytic figs germinate in the canopies of host trees as epiphytes [11]. After the seedlings become established, they descend aerial roots to the forest floor to anchor themselves around the host trees for additional physical support and to utilize soil water [12,13]. This is the hemi-epiphytic stage, and some species called “strangler figs” kill their host trees by strangling their trunk and finally, standing alone [11]. Thus, their seeds must be dispersed in the canopies of host trees and only arboreal and volant animals can be their potential seed dispersers. Seeds of hemi-epiphytic figs need to be dispersed in the host tree’s crown, but not everywhere in the crown. Several studies have indicated that a consistently moist condition is a primary requisite for germination, rather than light level [14–16], and the water retention ability of substrates at germination sites is the most important germination factor [15]. Common germination sites are tree forks, knotholes, axils of large branches, and broken or decomposed parts of the host trees [13,15,17,18], where suitable substrates are usually found. Due to the strict germination requirements, the epiphytic stage regulates hemi-epiphytic fig populations [19]. Therefore, defecation habits and behavior of seed dispersers directly affect their seed fates.

*Ficus* species are typical endozoochoric plants whose seeds are dispersed internally by frugivores. The germination rates of these seeds usually increase after being passed through animal digestive tracts, by separating pulp from fruits and seed scarification [20]. Additionally, fecal materials fertilize seedlings [20]. Some animals eat, destroy, or drop seeds beneath the maternal trees [21], but fig seed is too tiny to destroy, and most fig-eating animals can disperse its seeds [6,22]. However, the density of hemi-epiphytic figs is extraordinarily low [8], and there are many possible germination sites unsaturated in Bornean rainforests [8,23]. This indicates that most fig-eating animals may not disperse hemi-epiphytic fig seeds effectively.

Seed dispersal effectiveness (SDE) is the number of new adult plants produced by the activities of each seed disperser, and it is evaluated by both qualitative and quantitative components [24]. Quantity is the number of seeds dispersed, and quality is the probability that a dispersed seed produces a recruit. Quality is usually affected by the treatment of seeds in the mouth and gut and the seed deposition by the disperser [24]. Despite the ecological importance of hemi-epiphytic figs in the rainforests, seed dispersal systems by fig-eating animals under natural conditions remain unknown because of the difficulty in tracing the destiny of dispersed seeds in the canopy. Therefore, SDE has never been evaluated for hemi-epiphytic figs. In this study, we evaluated the SDE of potential seed dispersal agents of hemi-epiphytic figs on Borneo in terms of both quantity and quality. For the evaluation of quantity, we used two *Ficus* species, and for quality, we focused on several *Ficus* species. We also estimated seed dispersal distance of animal species. We selected three relatively large-sized (> 3 kg) arboreal and volant animals with over 50% of their diets consisting of fig fruits, to reduce the bias of fig consumption. Thus, we investigated binturongs *Arctictis binturong*, Mueller’s gibbons *Hylobates muelleri*, and helmeted hornbills *Rhinoplax vigil* as seed dispersal agents of hemi-epiphytic figs in Bornean lowland mixed dipterocarp forests.

## Material and methods

Permission to conduct the research was granted by the Sabah Biodiversity Centre of Sabah State Government. We were granted permission to capture and attach radio-collars to binturongs by the Sabah Biodiversity Council and the Sabah Wildlife Department (permission number: JKM/MBS.1000-2/2 JLD.4 (170), JKM/MBS.1000-2/2 JLD.5 (137), JKM/MBS.1000-2/2 JLD.7 (64)). Trapping and handling of the animals conformed to guidelines of the American Society of Mammalogists [25]. Research on gibbons and helmeted hornbills was non-invasive and involved direct observations. We kept certain distance from the animals so as not to disturb their behaviors.

### Study sites

We conducted this study at Danum Valley Conservation Area (Danum) and Maliau Basin Conservation Area (Maliau) in Sabah, north-eastern Borneo, from January 2013 to May 2014, and from February 2016 to June 2018, respectively. Danum (4°57ʹN, 117°48ʹE) is a 438 km^2^ protected zone, and 90% of this area consists of mature lowland evergreen dipterocarp forest [26]. Maliau (4°49′N, 116°54′E) is a 588 km^2^ protected zone. The study area was outside the basin in a selectively logged dipterocarp forest [27].

### Study animal species

Binturongs are the largest arboreal frugivorous carnivorans on Borneo. They weigh 6–10 kg [28] and fig fruits constitute nearly 90% of their diet [29]. Mueller’s gibbons are highly arboreal primates weighing 5–6.4 kg [28]. Fig fruits account for 1–56% of their diet, depending on the availability of other foods [30]. Helmeted hornbills are the largest hornbill species on Borneo; they weigh 2.6–3.1 kg [31] and nearly 100% of their diet constitutes fig fruits [32].

### Seed dispersal effectiveness

We calculated the SDE values of the three species and visualized them by SDE landscape [24]. The SDE landscape is a two-dimensional representation of the quantity and quality components. Because fig fruits include tiny and numerous seeds, we estimated number of fig seeds ingested by each species per day as the quantitative component. We assessed qualitative effectiveness based on germination rate and defecation microsite. Because we could not trace the post-germination destiny of dispersed seeds by the study animals, we used only defecation microsite data to estimate the one-year seedling survival rate as the qualitative component. To calculate the SDE value, we multiplied the estimated number of ingested fig seeds by each animal species by the estimated one-year survival probability.

### Quantitative component

First, we estimated the number of consumed fig fruits by observing the study animals feeding on hemi-epiphytic *Ficus* species (*Ficus benjamina* in Danum and *F*. *kerkhovenii* in Maliau) following the method of [33]. The relative fruit abundance in both periods was low. Visitation length was defined as the time interval between entry and exit for each individual of the three animal species during each visit, and feeding speed was recorded as the time spent to eat a fig fruit while the focal animal continuously consumed more than five fruits on the same branch with a precision of 0.1 s. We divided the mean visitation length by the mean feeding speed (time spent to eat a fig fruit) to estimate the total number of fig fruits eaten by each species per day. Then we estimated the ingested seed number by multiplying the mean seed number included in a fig fruit by the estimated total number of consumed fig fruits. These values were used as the quantitative component for evaluation of the total SDE.

At the Maliau site, we recorded only visitation length for binturongs because of the difficulty in observing feeding speed at night. The sample size for feeding speed and visitation length at each patch was too small for statistical analysis; thus, we pooled data from the two sites to estimate the probability of fig fruits consumed by each species after we confirmed that there were no significant differences in feeding speeds between the two sites (no data for binturongs at Maliau, Mueller’s gibbon p = 0.80, and helmeted hornbill p = 0.97). The crop and fig fruit sizes of these patches were similar; therefore, the effect of pooling the data was small.

Then, for the quantity component, we multiplied the mean seed number per fruit of the two hemi-epiphytic species, 148.7 [34], by the estimated number of consumed fig fruits by each animal species.

### Qualitative components

#### Germination rates

To test the effect of gut passage on seed germination, we planted defecated and control seeds derived from the same trees, as inferred from the average gut passage time. Because of the difficulties in determining the mother trees of ingested seeds, we included other growth forms besides hemi-epiphytes for analyses. We could not determine the mother trees of ingested seeds in gibbon feces, so we used data from a previous study [35] on hybrid Mueller’s gibbons (*Hylobates muelleri* × *agilis*). We planted fig seeds of three species collected from binturong feces (hemi-epiphytes *F*. *forstenii* (two individuals) and *F*. *stupenda*, and climber *F*. *punctata*) and two species collected from helmeted hornbill feces (hemi-epiphyte *F*. *benjamina* and climber *F*. *trichocarpa*). For hybrid Mueller’s gibbons, we referred to data for four hemi-epiphytes (*F*. *crassiramea*, *F*. *kerkhovenii*, *F*. *sumatrana*, *F*. *stupenda*) and a climber (*F*. *sinuata*) from the previous study [35]. It was not practicable to collect feces from all three study animals that fed in the same trees. We extracted 100 seeds from the feces or figs and placed them in plastic nursery bags filled with forest soil within six hours of collection. We recorded germination weekly for eight weeks. We assessed the effect of gut passage on germination rates using Fisher’s exact test.

#### Seed dispersal microsites

We located the microsites where we found feces of the study animals by individually tracking them after they left the feeding patches or when we found them by chance. Because of the nocturnality of binturongs, we radio-tagged three [29] and recorded their seed dispersal microsites at their feeding and sleeping sites by following them. We also recorded defecation microsites of non-radio-tagged binturongs at their feeding and sleeping sites. We used the single-rope technique to access the defecation sites in the canopy [36]. The microsites were classified into five categories: tree fork, branch, epiphytic mat, foliage, and forest floor. We attempted to record survival of the dispersed seeds at each site, but all died out or became inaccessible during the study period. Therefore, we used seedling survival rates per microsite after one year, based on [15]. The one-year seedling survival rates in tree forks, epiphyte clumps, branches, and knotholes were 3.7, 7.6, 4.7, and 13.9%, respectively [15]. We assumed that not all seeds deposited on the forest floor and foliage would survive because the germination conditions were unsuitable, or rains washed the seeds away to the forest floor [23]. The zero value for the survival rate was substituted with 0.03% to minimize the Type I error [37]. We calculated the frequency of finding feces at each microsite and estimated the seedling survival rate per study animal by multiplying the one-year seedling survival rate at each microsite. These values were used as the qualitative component for the evaluation of SDE.

We recorded establishment microsites of hemi-epiphytic fig saplings to evaluate resemblance between sapling position and seed dispersal microsite per study animal. We calculated the Bray-Curtis dissimilarity index among microsites. Then, we used these indices to ordinate the units by non-metric multidimensional scaling (NMDS) using package “vegan” in R version 3.4.3 [38].

### Dispersal distance

We estimated the seed dispersal kernels [39] per study animal by combining the distance of animals from the origin at each hour and seed deposition time. The moving distance of animals at each hour was based on direct following of binturongs by radio-tracking (see [29]) and gibbons (S1 Table) in the study areas. Because of the small number of successful followings of helmeted hornbills from the feeding to defecation sites, we used hourly movement data of a similarly sized hornbill, great hornbill *Buceros bicornis*, in a mosaic of seasonally evergreen forest and grassland in Thailand (S1 Table) [40]. We fitted the seed deposition times with the Gamma distribution, which was based on the mean and variance of empirical gut retention time data of a hybrid Mueller’s gibbon (*Hylobates muelleri × agilis*) [35] and binturongs [41] (S1 Table). For helmeted hornbills, we used data of a similarly sized Sulawesi’s hornbill species, knobbed hornbill *Rhyticeros cassidix* [42] (S1 Table). We randomly drew moving distance and seed deposition times for 1000 repetitions to estimate dispersal kernels.

## Results

### Quantitative component

**Fig 1:**
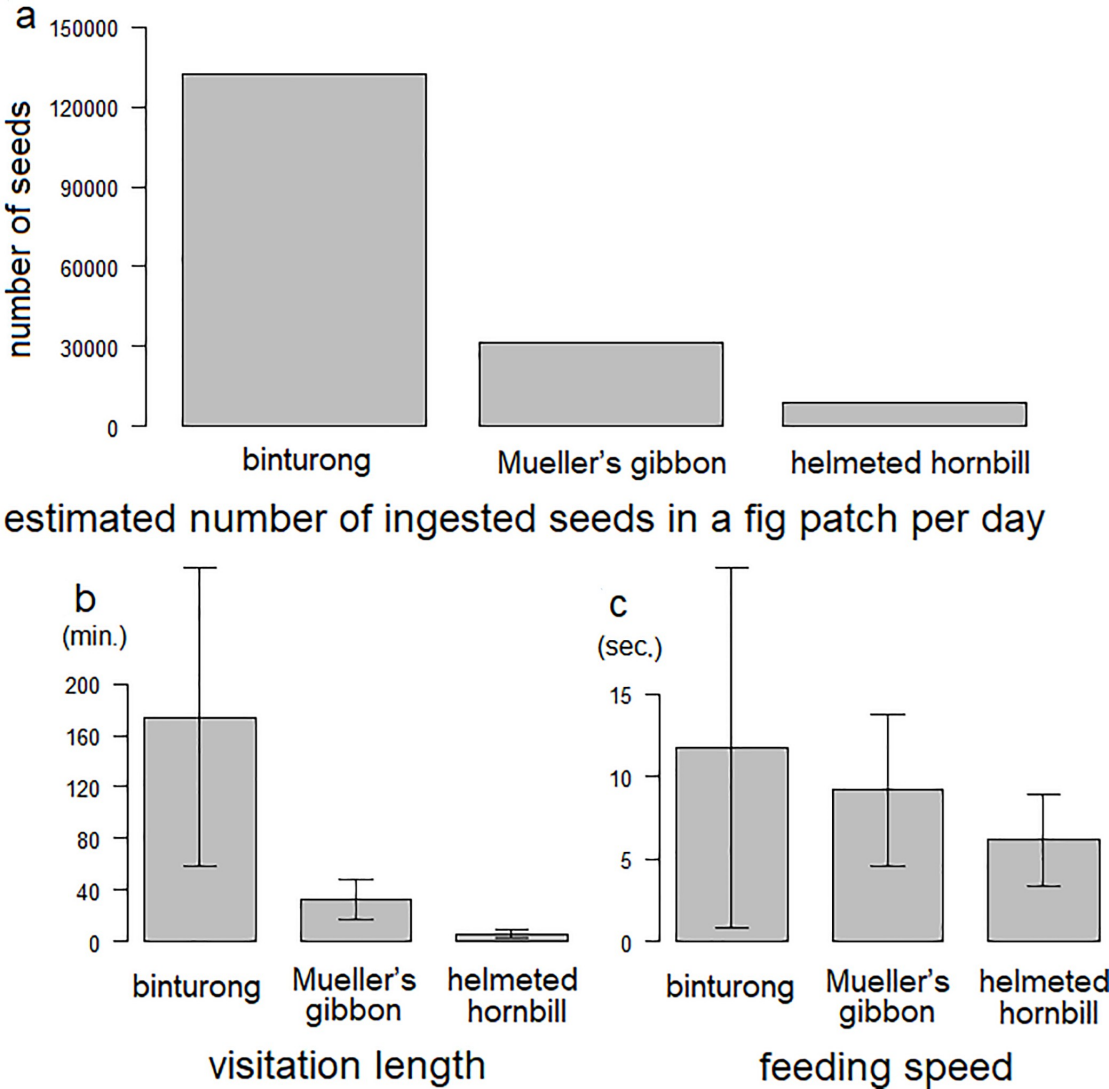
Estimated number of ingested fig seeds per day (1a), visitation length (1b), and feeding speed (1c) of binturongs, Mueller’s gibbons, and helmeted hornbills.

We conducted comprehensive field observations at two hemi-epiphytic fig patches: *F*. *benjamina* at Danum for six continuous days, totaling 108 hours [33] and *F*. *kerkhovenii* at Maliau for four continuous days, totaling 56 hours. In both patches, binturongs, Mueller’s gibbons, and helmeted hornbills fed on whole fig fruits, while several consumers, such as long-tailed macaques, pigeons, squirrels, and barbets often partially consumed fig fruits. The mean visitation length (minutes ± standard deviation) per day for binturongs, gibbons, and helmeted hornbills was 207.2 ± 71.6 (n = 9), 47.5 ± 12.7 (n = 4), and 6.0 ± 3.4 (n = 5), respectively. The mean feeding speed (seconds/fig fruit) of binturongs, gibbons, and helmeted hornbills was 11.69 ± 10.91 (n = 5), 9.21 ± 4.64 (n = 8), and 6.19 ± 2.81 (n = 5), respectively; the estimated number of fig fruits consumed in a fig patch per day was 1063.47, 309.45, and 58.16, respectively; and the estimated ingested seed number in a fig patch per day was 132640.4, 31554.1, and 8713.8, respectively (Fig 1). Binturongs ingested substantially more fig seeds in the same trees than gibbons and helmeted hornbills.

### Qualitative component

#### Germination rates

The germination rates of all species except *F*. *stupenda* were significantly higher after being defecated by binturongs (all p < 0.01, Table 1). *Ficus benjamina* seeds showed a reduced germination rate after being defecated by a helmeted hornbill (p < 0.01), but that of *F*. *trichocarpa* was higher than the control (p < 0.01). All the fig species germinated significantly faster after being defecated by both animals (Table 1).

**Table 1:** Germination tests for the ingested and control seeds.

Study Animals	*Ficus* species	Life form^a^	Germination rate (%)		Effect (p-value)^b^	Days to germination	
			ingested	control		ingested	control
Binturong	*F*. *forstenii*1	H	70	47	+ (<0.01)	5	6
	*F*. *forstenii*2	H	43	15	+ (<0.01)	–	–
	*F*. *punctata*	C	53	2	+ (<0.01)	4	17
	*F*. *stupenda*	H	19	30	n.s.	3	5
Gibbon (*Hylobates muelleri* × *agilis*) data from [35]	*F*. *crassiramea*	H	0	0.3	n.s.	–	–
	*F*. *kerkhoevenii*	H	22	0	+	1	–
	*F*. *sinuata*	C	93	17	+	5	13
	*F*. *sumatrana*	H	47	0	+	3	–
	*F*. *stupenda*	H	43	11	+	4	4
Helmeted hornbill	*F*. *benjamina*	H	11	46	- (<0.01)	8	10
	*F*. *trichocarpa*	C	14	2	+ (<0.01)	9	21

^a^ H: hemi-epiphyte, C: climber

^b^ whether animals enhance (+) or hinder (–) germination of each *Ficus* species

#### Seed dispersal microsites

We located 37, 56, and 17 seed dispersal microsites for binturongs, Mueller’s gibbons, and helmeted hornbills, respectively. Binturongs disproportionally defecated in the canopy: tree forks (47.2%), branches (30.6%), epiphytic mats (19.4%), and forest floor (2.8%). The estimated one-year seedling survival rates (%) at each microsite were 0.017, 0.014, 0.015, and 0.0003, respectively. Most gibbon feces were found on the forest floor (96.4%) and on foliage in the understory (3.6%). The estimated one-year seedling survival rate of both sites was 0.0003. Helmeted hornbill feces were deposited on the forest floor (58.8%), understory foliage (29.4%), and branches (11.8%). The estimated one-year seedling survival rates at each site were 0.0003, 0.0003, 0.014, respectively. All three study animals defecated in the canopy level, but only binturongs deposited their feces on microsites in the canopy. They defecated on relatively flat tree surfaces, such as tree forks, large epiphytes, and large branches; and they often rubbed their feces onto the surface of the defecation sites. Gibbons and helmeted hornbills let the feces drop down from the canopy.

**Fig 2:**
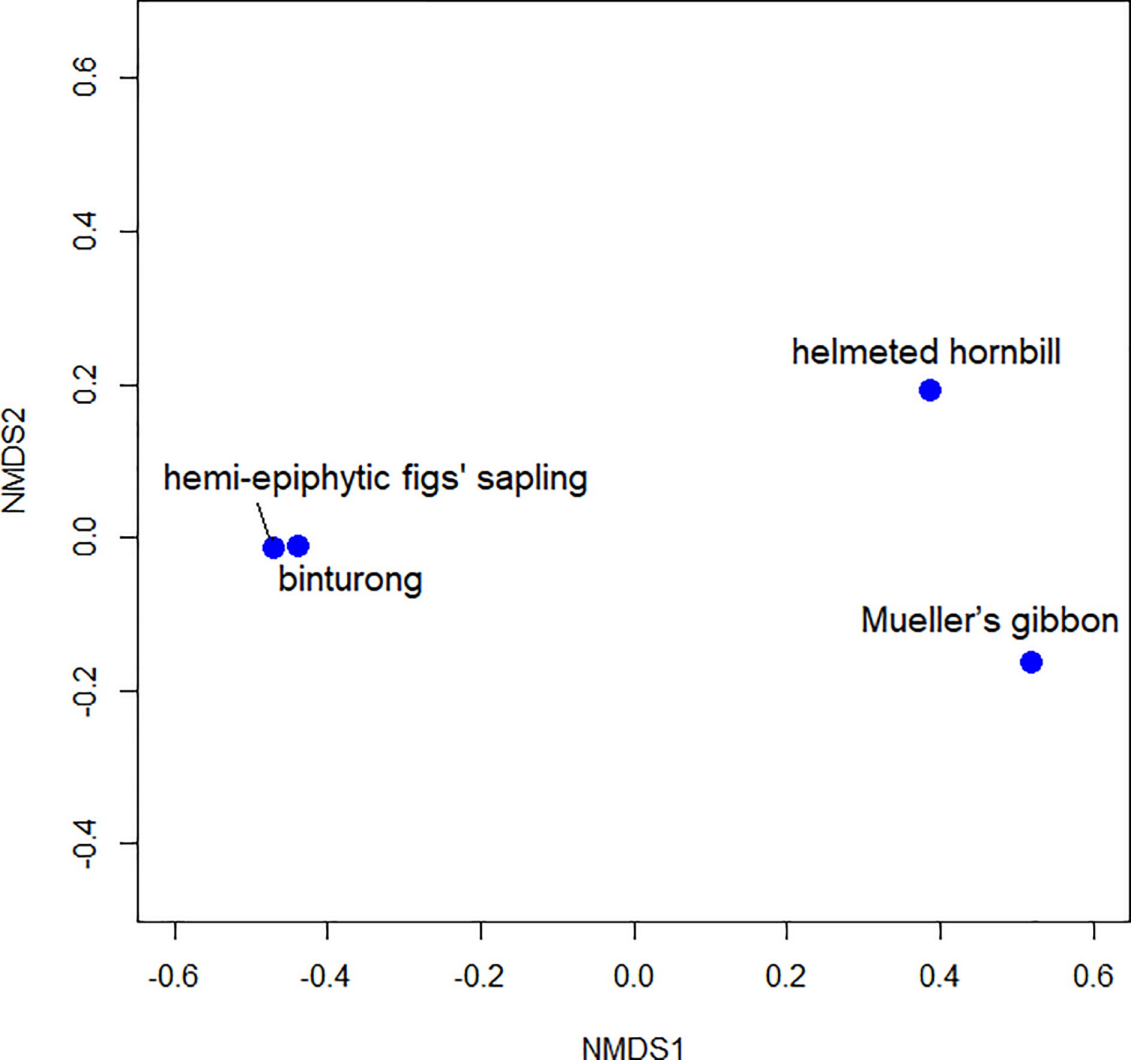
Non-metric multidimensional scaling (NMDS) ordination of microsites generated by binturongs, Mueller’s gibbons, helmeted hornbills, and hemi-epiphytic fig saplings.

Most hemi-epiphytic fig saplings (n = 45) were encountered on tree forks (73.3%), followed by branches (22.2%), knotholes (2.3%), and epiphytic mats (2.3%). The NMDS ordination showed that binturong dispersal microsites coincided with fig establishment sites; and gibbon and hornbill dispersal microsites were neither similar to fig establishment sites nor to each other (Fig 2). The stress value of the NMDS ordination was < 0.01.

### Seed dispersal effectiveness

**Fig 3:**
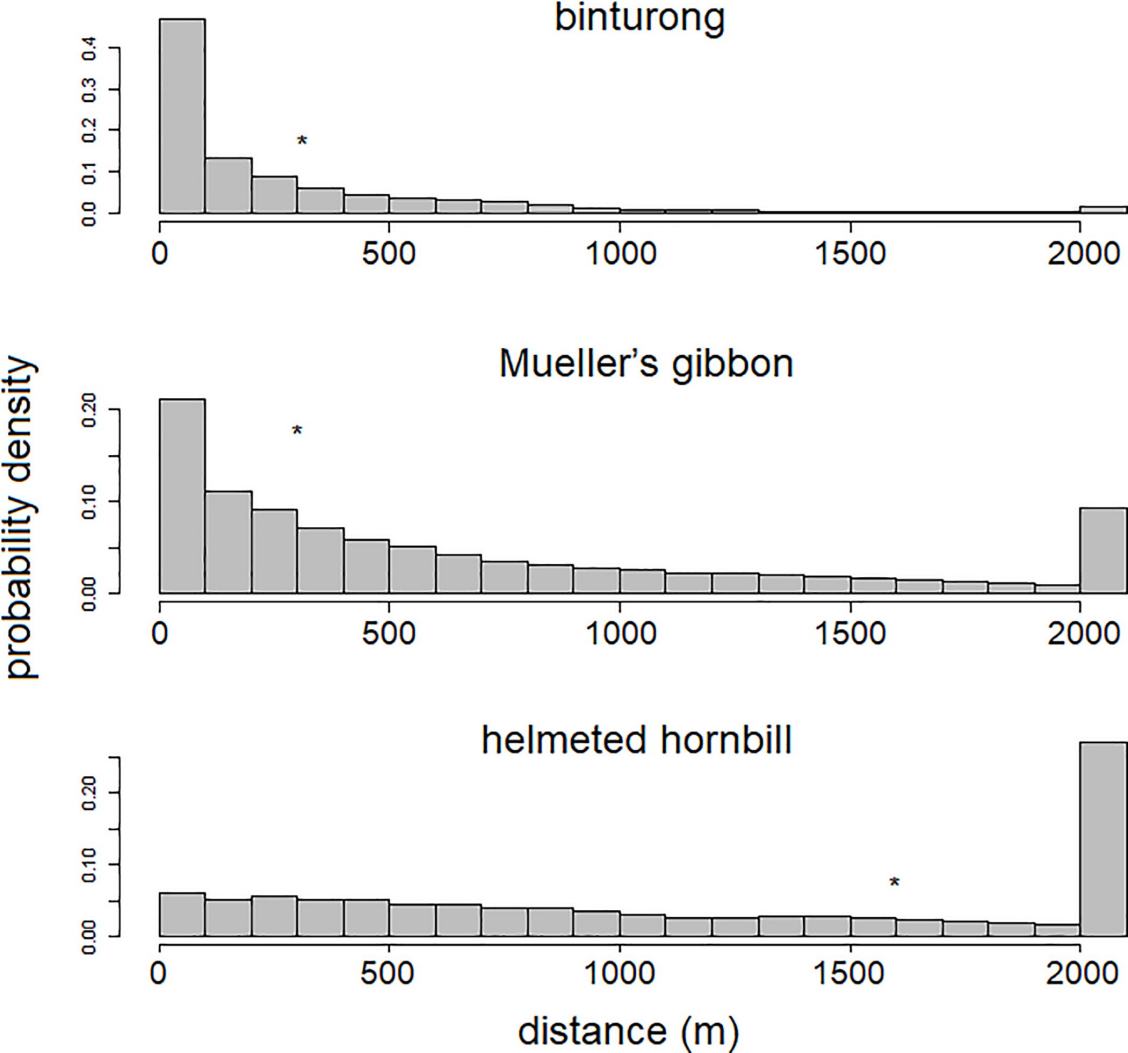
The total seed dispersal effectiveness (SDE) of binturongs, Mueller’s gibbons, and helmeted hornbills across their defecation microsites, per quantitative (estimated number of ingested seeds per day) and qualitative (estimated one-year seedling survival rate based on defecation microsite data) components. The elevational contours depict the isoclines of SDE. The numbers on the apex of each isocline indicate SDE values. SDE values to the right and above have greater effectiveness.

The SDE values of binturongs at each dispersal microsite—tree fork, branch, epiphytic mat, and forest floor—were 2317.5, 1904.9, 1960.1, and 3.9, respectively. Those of Mueller’s gibbons at foliage and forest floor were 0.9 and 0.9, respectively, and those of helmeted hornbills at branch, foliage, and forest floor were 120.5, 0.3, and 0.3, respectively. The total SDE values differed considerably per study animal and seed dispersal microsite (Fig 3). The binturongs’ effectiveness was the highest among the study animals when they defecated at tree forks, branches, and epiphytic mats. Although the SDE values of helmeted hornbills were low, the qualitative effectiveness was high when their feces stuck to branches. The SDE of the three species showed the same value when they dispersed seeds on the forest floor and foliage.

### Dispersal distance

**Fig 4:**
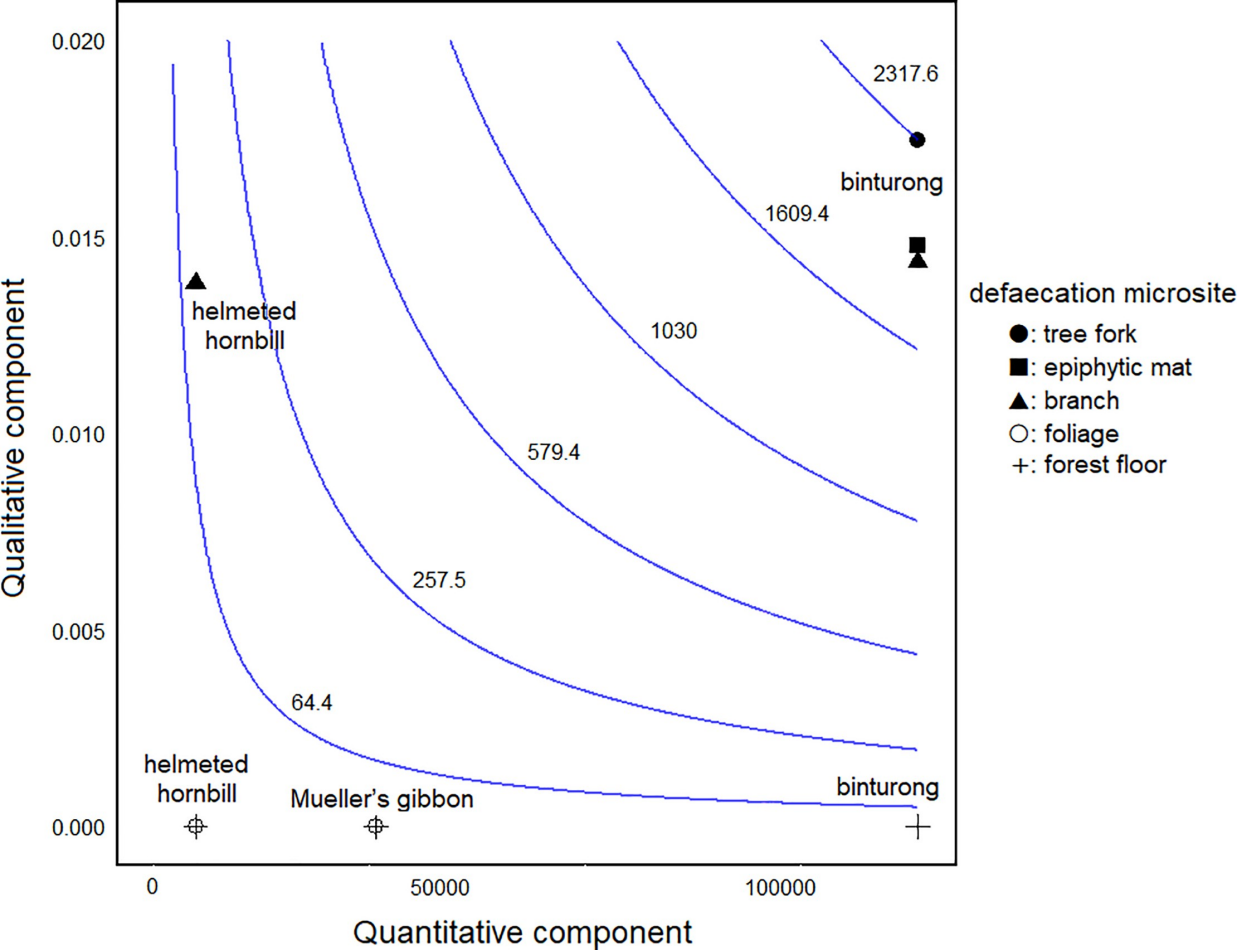
Seed dispersal kernels of binturongs, Mueller’s gibbons, and helmeted hornbills. Asterisks represent the mean values.

Seed dispersal kernels generated by binturongs ranged from 0 to 6105.3 m, with a mean distance of 342.7 ± 505.5 m (mean ± standard deviation). Approximately 16.3% of ingested seeds will be dispersed within 10 m of the parent trees, and 44.1% and 9.1% of the seeds would be dispersed within 100 m and more than 1 km, respectively (Fig 4). Seed dispersal kernels generated by Mueller’s gibbons ranged from 20.3 to 1893.2 m, with a mean distance of 338.8 ± 197.2 m. They would disperse all ingested seeds more than 10 m from the parent trees, and 5.6% and 0.8% of seeds would be dispersed within 100 m and more than 1 km, respectively (Fig 4). That of helmeted hornbills ranged from 0.9 to 10,707.6 m, with a mean distance of 1611.8 ± 1569.1 m. They would disperse 0.44% of the ingested seeds within 10 m from the parent trees, and 5.4% and 53.3% of the seeds would be dispersed within 100 m and more than 1 km, respectively (Fig 4).

## Discussion

### Quantity

The number of fig seeds ingested in the same patch differed considerably among the three frugivorous species. Binturongs consumed by far the largest number of fig fruits in the feeding patch followed by Mueller’s gibbons and helmeted hornbills. However, this does not indicate that a binturong is a quantitatively effective seed disperser at the population level of *Ficus* species because it tends to stay for a long time at a single feeding patch [29]. At the individual fig level, binturongs had more opportunities than the other two species to disperse hemi-epiphytic fig seeds. At the fig population level, animals feeding at several fig patches in a day, such as gibbons and hornbills (M Nakabayashi, pers. obs. and [42]) would contribute more than those, such as binturongs, that visited a few patches in a day.

### Quality

#### Germination rate

Given that all three animal species ejected viable seeds to an extent, gut passage by the three animal species did not adversely affect seed germination. A previous study reported rapid and high germination of a hemi-epiphytic species *Ficus benghalensis* after passing through the bird digestive tract [43]. However, the benefit of gut passage for hemi-epiphytic figs in our results was variable among the *Ficus* species and probably individuals, and it is unclear compared to that for climber species (Table 1). This study used relatively large animals, so gut passage may have a different effect than with smaller birds. The obvious positive effect of gut passage on climbers might be attributed to their growth pattern. Generally, tested climber figs germinate and establish on the forest floor [44], and they need to climb tall trees and get to the high-light canopy rapidly [45]. Therefore, the rapid and high germination of ingested seeds are beneficial for their survival and growth. However, these benefits might not be useful for hemi-epiphytic figs if ingested seeds hit unsuitable sites. Therefore, the most beneficial point of ingestion for the seeds of hemi-epiphytic figs could be the existence of viable seeds, rather than high and rapid germination. Ingestion by all three animal species fulfils this requirement.

#### Dispersal microsite

This is the most decisive component of survival for hemi-epiphytic figs [19]. Of the dispersed microsites generated by the three animal species, only tree forks, epiphyte clumps, and branches are potential establishment microsites [15]. Our results show that binturongs disperse fig seeds into suitable establishment sites for hemi-epiphytic figs. Binturongs have difficulty in digesting fruits efficiently because of their morpho-physiological constraints [41, 46]: their feces are watery, making it easier to stick to tree bark. The reason for the binturongs’ defecatory habits is unclear; however, given that seed deposition determines the fate of hemi-epiphytic figs, these habits are notably beneficial for fig survival. Binturongs feed at various heights, from the understory to upper canopy strata [47], distributing seeds to all canopy layers. Moreover, they frequently rest on large and relatively horizontal branches of dipterocarps (M Nakabayashi, pers. obs. and [48]), which are the major host plants of hemi-epiphytic figs on Borneo [8]. These behaviors largely contribute to an increased probability of establishment success.

To date, reliable directed dispersal of hemi-epiphytic figs has not been thought to occur in Bornean rainforests [23], but the present study shows that it occurs via binturongs. Despite their high reproductivity and many potential seed dispersal agents [6], only 0.01% of seeds are dispersed in suitable sites [23] and 1.3% of seedlings will survive to one year [15]. This rate is strikingly lower than that of non-hemi-epiphytic figs [49], even though hemi-epiphytic figs are tolerant to severe conditions, such as water shortage, compared to non-hemi-epiphytic figs [50]. One of the reasons for its low successful dispersal could be the difficulty in seed dispersal in mature Bornean rainforests with developed forest strata [51]. The opportunity of seeds to reach specific microsites for establishment in vertically stratified forests is likely much lower than in forests with less developed canopy structures. In the uneven canopy of Bornean rainforests, defecating directly at the establishment sites is the most reliable dispersal method, compared to scattering feces from the air or upper canopy. Hemi-epiphytic fig species account for over 70% of the *Ficus* species that binturongs feed on in Bornean rainforests [29]. Therefore, eventually binturong behaviors lead to directed seed dispersal [52] of hemi-epiphytic figs.

### Total SDE

In the present study, only three large frugivorous animals were assessed for their effectiveness as seed dispersers of hemi-epiphytic figs. Among them, binturongs were the most effective disperser in both quality and quantity. We focused on different hemi-epiphytic *Ficus* species for quantity and quality evaluations, but these species are in the same subsection of the same subgenus [1] and have almost same seed sizes [M Nakabayashi unpubl. data]. Therefore, the effect of gut treatment on germination for different *Ficus* species would be small. Although binturongs and Mueller’s gibbons were more quantitatively effective than helmeted hornbills, the total SDE was the same when they defecated at unsuitable sites. Although helmeted hornbills were not quantitatively effective when compared to the other two species, the total SDE increased when the ingested seeds hit suitable microsites. These results indicate that quality is more critical than quantity for hemi-epiphytic figs. The degree of effects of quality and quantity components on total SDE differs with seed dispersers, plants, environment, and also methodology [53,54]. In other areas and other plants, dispersers usually have higher effectiveness either quantitatively or qualitatively [53,54], whereas binturongs showed high values in both components. The seed deposition microsite is the most critical factor for survival of hemi-epiphytic figs, so total SDE was biased toward quality.

### Dispersal distance

All three animal species potentially disperse seeds beyond the tree crowns. Given that mechanical seed dispersal distance is generally within 10 m of the tree [55], Mueller’s gibbons and helmeted hornbills provide higher opportunities for long distance dispersal—over 100 m—than binturongs. The former two species showed similar dispersal patterns as they rarely dispersed seeds within 100 m, but almost no ingested seeds of Mueller’s gibbons were dispersed over 1 km, whereas over 50% of those of helmeted hornbills were. Because binturongs frequently rest at the feeding sites [29], some of the dispersed seeds accumulate in the same places (M Nakabayashi, pers. obs.) and 16% of seeds are dispersed within 10 m of the parent trees. However, they frequently change feeding and rest sites [29,56], thus scattering these seeds across their ranges and 55% of seeds are dispersed over 100m. Mueller’s gibbons and helmeted hornbills dispersed seeds over 100 m with higher probability than binturongs, and helmeted hornbills were the only reliable dispersers of over 1 km among the three species.

Generally, longer distance dispersal is exhibited by animals having larger home-range size [57], but this tendency is likely to depend on the behavior of animals. Although binturongs have much larger home-range sizes (1.5–4.2 km^2^; [29]) than Mueller’s gibbons (0.3 km^2^; [30]), the mean dispersal distance of the two species are almost the same. This difference is relevant to the binturongs’ feeding strategy. They tend to stay at the same feeding patch for a long duration [29], so their daily movement distance is much shorter (228 m; [29]) than that of Mueller’s gibbons (1113 m; [30]). Meanwhile, helmeted hornbills have the largest dispersal distance because they might have the largest home-range based on data for similarly sized hornbills (4.1–34.9 km^2^; [39]).

### Dynamic of hemi-epiphytic figs

Binturongs are effective seed dispersers for hemi-epiphytic figs, but they are not the only ones. In this study, we demonstrated that ingested fig seeds by all the study animals were viable, and most were notably transported away from the parent trees. Although helmeted hornbills were less effective than binturongs, some of their feces stuck to the potential germination sites. Fig seeds in knotholes showed the highest survival rates among the potential germination microsites [15]. When knotholes were used for breeding nests [58], some seeds survived fortuitously on the lip of the cavity [59], while most were dispersed to the forest floor around the nest [60]. Considering that over 50% of ingested seeds would be dispersed to more than 1 km, they are critically important long-distance seed dispersal agents for promoting gene flow among populations [55]. Another closely related species to binturongs, the small-toothed palm civet *Arctogalidia trivirgata*, also exhibits similar defecatory habits (M Nakabayashi, pers. obs.). Because they usually consume hemi-epiphytic figs [47], they are also potentially effective seed dispersers. Secondary seed dispersal by ants [61,62] should also be considered; although, the most frequently observed canopy ant, *Pheidole*, could also be a seed predator in Borneo [62]. These animals may increase the probability of seeds hitting suitable establishment sites.

A single fig tree produces a massive amount of seeds per single fruiting event (400,000–13,000,000), maximizing the probability of establishment [23]. Therefore, most fig-eating animals can be potential seed dispersal agents when they defecate some viable seeds [6, 22]; though, over 50% of hemi-epiphytic fig seeds fell under the parent trees [23]. Fig fruit size demonstrates the importance of large-bodied effective seed dispersers of hemi-epiphytic figs, such as binturongs and helmeted hornbills. They feed on fig fruits of a broad size range (1–7 cm in diameter; [29,63]), including *F*. *stupenda*, one of the largest hemi-epiphytic figs on Borneo [1]. *Ficus* species that bear large fruits on Borneo are usually climbers and trees that germinate on forest floors, but there are several hemi-epiphytic figs that bear large fruits, such as *F*. *cucurbitina*, *F*. *dubia*, *F*. *stupenda*, and *F*. *xylophylla* [1]. Fruit bats and birds are important seed dispersers, especially on oceanic islands because they carry seeds from continental lands [64]. However, because large hemi-epiphytic fig seeds are surrounded by thick and hard flesh (inflorescence), most birds and bats do not consume whole fig fruits but pick or gnaw the flesh and leave the seeds untouched (M Nakabayashi, pers. obs. and [65]). Indeed, there are no hemi-epiphytic figs producing large fruits on newly formed volcanic islands, where birds and bats are the main seed dispersal agents [64], and these figs are poorly dispersed in the Bornean rainforest that lacks several large frugivores [10]. Binturongs and helmeted hornbills swallow these large fig fruits together with seeds (M Nakabayashi pers. obs.). Moreover, given that species bearing large fig fruits usually occur at high positions in the canopy [8] where more severe conditions of water shortage for epiphytes are present, these species could be more dependent on seed deposition on suitable microsites. Thus, they significantly contribute to the survival of hemi-epiphytic figs that bear large fruits.

Gibbons and hornbills are generally the most important seed dispersal animals in Southeast Asian rainforests [21,22]. However, most studies assessing their SDE have used ground-based plants [34,66]. This study demonstrated that, compared to terrestrial plants, successful seed dispersal of hemi-epiphytic figs, which requires strict conditions for seedling establishment, by these frugivores is aleatory and rare, especially in gibbons. They largely depend on figs for their diet [30], but most previous studies have identified *Ficus* species only to the genus level. Therefore, their dependence on hemi-epiphytic species is unclear. Seed deposition on the forest floor is optimal for most non-hemi-epiphytic *Ficus* species, so gibbons definitely contribute to the seed dispersal of these *Ficus* species. Directed seed dispersal to specific microsites in the canopy is limited to specific animal species. Therefore, it may become one of the main factors regulating low-success hemi-epiphytic fig recruitment in Bornean rainforests.

Our study focused on only three relatively large frugivores, and their SDE at the population and community levels remains inconclusive. Unfortunately, the important seed dispersers of these hemi-epiphytic figs are listed as Vulnerable (binturongs [67]) and Critically Endangered (helmeted hornbills [68]). As populations of both species are decreasing [67, 68], if they become extinct, seed dispersal opportunities for these figs would be drastically decreased, subsequently affecting other animals that feed on them and eventually local ecosystems. More efforts are urgently needed to implement practical conservation policies for these important animals. Additionally, more studies are needed to understand the reproductive systems of keystone plants, such as hemi-epiphytic figs, in tropical rainforests.

## Supporting information

S1 Table Moved distance (m) from the origin at each hour and empirical gut retention time (hour) of binturongs, gibbons, and hornbills. a: data of the gut retention time is from [41]. b: data of the gut retention time is from [35] (a *Hylobates muelleri* × *agilis*). c: data of the moved distance is from [40] (a *Buceros bicornis*), and that of gut retention time is from [42] (a *Rhyticeros cassidix*)
(DOCX)

S2 Table Raw data of visitation length (min.) and feeding speed (sec.). (XLSX)

S3 Table Raw data of seed dispersal microsites and fig establishment sites. (CSV)

S4 Table Raw data of moved distance from the origin at each hour (m). (CSV)
