# Supplementary material for: Limited directed seed dispersal in the canopy as one of the determinants of the low hemi-epiphytic figs’ recruitments in Bornean rainforests
Source: PLoS One. 2019 Jun 13;14(6):e0217590. doi: 10.1371/journal.pone.0217590 (PMC6564369; doi:10.1371/journal.pone.0217590)
Supplement: S1 Table — a: data of the gut retention time is from [41]. b: data of the gut retention time is from [35] (a Hylobates muelleri × agilis). c: data of the moved distance is from [40] (a Buceros bicornis), and that of gut retention time is from [42] (a Rhyticeros cassidix) (DOCX) [file pone.0217590.s001.docx]

Table S1. Moved distance (m) from the origin at each hour and empirical gut retention time (hour) of binturongs, gibbons, and hornbills.

| Animal species | | Binturong^a^ | Gibbon^b^ | Hornbill^c^ |
| --- | --- | --- | --- | --- |
| Moved distance  (m) | mean ± SD | 50.3 ± 76.4 (n = 63) | 27.5 ± 33.4 (n = 63) | 1148.8 ± 1125.3 (n = 23) |
|  | max. | 328.2 | 148.6 | 5734.5 |
|  | min. | 0 | 1.2 | 301.8 |
| Gut retention time (hour) | mean ± SD | 6.5 ± 0.3 | 27.8 ± 10.7 | 1.4 |
|  | max. | 3.3 | 15.1 | 1 |
|  | min. | 9.3 | 50.1 | 1.9 |

a: data of the gut retention time is from [41]

b: data of the gut retention time is from [35] (a *Hylobates muelleri* × *agilis*)

c: data of the moved distance is from [40] (a *Buceros bicornis*), and that of gut retention time is from [42] (a *Rhyticeros cassidix*)
